# Titrimetric and Spectrophotometric Assay of Oxcarbazepine in Pharmaceuticals Using N-Bromosuccinimide and Bromopyrogallol Red

**DOI:** 10.1155/2011/138628

**Published:** 2011-07-18

**Authors:** Nagaraju Rajendraprasad, Kanakapura Basavaiah, Kanakapura B. Vinay

**Affiliations:** Department of Chemistry, University of Mysore, Manasagangothri, Mysore-570 006, Karnataka, India

## Abstract

Titrimetric and spectrophotometric methods are described for the determination of oxcarbazepine (OXC) in bulk drug and in tablets. The methods use N-bromosuccinimide (NBS) and bromopyrogallol red (BPR) as reagents. In titrimetry (method A), an acidified solution of OXC is titrated directly with NBS using methyl orange as indicator. Spectrophotometry (method B) involves the addition of known excess of NBS to an acidified solution of OXC followed by the determination of the unreacted NBS by reacting with BPR and measuring the absorbance of the unreacted dye at 460 nm. Titrimetry allows the determination of 6–18 mg of OXC and follows a reaction stoichiometry of 1 : 1 (OXC : NBS), whereas spectrophotometry is applicable over the concentration range of 0.8–8.0 *μ*g mL^−1^. Method B with a calculated molar absorptivity of 2.52 × 10^4^ L mol^−1^ cm^−1^ is the most sensitive spectrophotometric method ever developed for OXC. The optical characteristics such as limits of detection (LOD), quantification (LOQ), and Sandell's sensitivity values are also reported for the spectrophotometric method. The accuracy and precision of the methods were studied on intraday and interday basis. The methods described could usefully be applied to routine quality control of tablets containing OXC. No interference was observed from common pharmaceutical adjuvants. Statistical comparison of the results with a reference method shows an excellent agreement and indicates no significant difference in accuracy and precision. The reliability of the methods was further ascertained by recovery studies in standard addition procedure.

## 1. Introduction

Oxcarbazepine (OXC), (chemically known as 10,11-dihydro-10-oxo-5H-dibenzo[b,f]azepine-5-carboxamide), is a novel antiepileptic drug, which was developed as a second generation and a follow-up compound to carbamazepine. Clinically, it has been used to treat several types of epilepsy [1–3] and bipolar disorders [4].

The ever-increasing use of OXC in pharmaceutical formulations has necessitated its determination as a matter of foremost importance. OXC is not official in any Pharmacopoeia. Various analytical methods such as HPLC [5–8], HPTLC [9], GC [8], microemulsion electrokinetic chromatography [10], capillary electrokinetic chromatography [11], voltammetry [12, 13] and capillary electrophoresis [14] have been reported for the determination of OXC in pharmaceuticals. However, these methods involve the use of expensive instruments which are not available at most quality control laboratories in developing and underdeveloped nations.

Titrimetry is still widely used in analytical chemistry for its superior speed and simplicity with little sacrifice in accuracy and precision. OXC is present in relatively large amounts (150 to 600 mg per tablet) in pharmaceutical formulations. The simplicity and rapidity of titrimetry is more advantageous especially for macroanalysis over any instrumental methods. Since there is no stringent conditions to be maintained, more precise and accurate results are obtained from titrimetry. Because of one or the other advantage of titrimetry, it is widely applicable for the assay of many pharmaceutical compounds in many Pharmacopoeias. On the other hand, the spectrophotometric technique continues to be used in the assay of different classes of drugs in pure and in pharmaceutical formulations [15–18].

Despite being one of the most widely used antiepileptic drugs, no titrimetric method has been reported for OXC, and even a few visible spectrophotometric methods reported [19–21] suffer from one or another disadvantage. Of the two methods reported by Gandhimathi and Ravi [19], the first method uses Folin-Ciocalteu's (F-C) reagent in alkaline medium in which blue chromogen measured at 760 nm, whereas the second method involves addition of a fixed volume of 3-methyl-2-benzothiazolinone hydrazine hydrochloride (MBTH) after treating of OXC with ferric chloride followed by the measurement of absorbance at 456 nm. The methods obey Beer's law over the linear range of 5–30 and 10–50 *μ*g mL^−1^ for F-C and MBTH methods, respectively, and the corresponding molar absorptivity values are 8.06 × 10^3^ and 3.126 × 10^3^ L mol^−1^ cm^−1^. In another report [20], OXC has been determined using iron(III) chloride and potassium hexacyanoferrate(III). The method is based on the reduction of iron(III) ions to iron(II) ions by drug, which in presence of hexacyanoferrate(III) produces green coloured chromogen measurable at 770 nm. This method is applicable over the concentration range of 4–28 *μ*g mL^−1^ OXC with the molar absorptivity value of 4.63 × 10^3^ L mol^−1^ cm^−1^. The yellow chromogen with an absorption maximum at 430 nm formed by the reaction of OXC with methanolic KOH in DMSO medium as the basis for the assay of OXC (linear range 1.0–7.0 *μ*g mL^−1^; molar absorptivity 1.21 × 10^4^ L mol^−1^ cm^−1^) has been reported by Sathish and Nagendrappa [21].

All the four reported spectrophotometric methods are less sensitive besides employing either organic solvent medium or heating step. 

In the present paper, we report one direct titrimetric (method A) and one indirect spectrophotometric (method B) methods using NBS as brominating agent in sulphuric acid medium for the assay of OXC. In titrimetry, the acidic solution of OXC was titrated with NBS in sulphuric acid medium. In spectrophotometry, for the first time, bromopyrogallol red (BPR) has been used to determine the unreacted NBS after completion of the reaction between OXC and NBS in sulphuric acid medium. The proposed methods are simple, highly accurate, and can be readily applied to the determination of OXC in bulk drug and in tablets.

## 2. Experiment

### 2.1. Apparatus

A Systronics model 106 digital spectrophotometer with 1 cm path length matched quartz cells was used to record the absorbance values.

### 2.2. Reagents and Standards

All chemicals used were of analytical reagent grade. Distilled water was used throughout the investigation.

Acetic acid (2 : 3 and 1 : 9 v/v) and sulphuric acid (10 M) were prepared by diluting appropriate volumes of their concentrated acids (99% glacial acetic acid and 98% sulphuric acid -both from Merck, Mumbai, India) with water. Two brands of tablets namely, Trioptal-300 (Novartis India Ltd, Mumbai, India) and Oxetol-600 (Sun Pharmaceuticals, Sikkim) were purchased from local commercial sources.

#### 2.2.1. N-Bromosuccinimide (0.01 M and 120 *μ*g mL^−1^)

An approximately 0.01 M solution was prepared by dissolving an accurately weighed quantity of NBS (Loba Chemie Ltd, Mumbai, India, assay 99) in water with the aid of heat. The solution was cooled to room temperature, standardized iodometrically, kept in an ambered coloured bottle, and stored at 4°C. For method B, NBS solution after standardization was diluted with water to get a working concentration of 120 *μ*g mL^−1^.

#### 2.2.2. Bromopyarogallol Red (BPR) (0.03%)

An aqueous 0.03% BPR solution was prepared daily afresh by dissolving an accurately weighed 30 mg of the dye (Loba Chemie, Mumbai, India, purity 99%) in 10 mL of methanol in a 100 mL volumetric flask, and the volume was made upto the mark with water.

#### 2.2.3. Standard OXC Solution

A stock standard solution containing 2 mg mL^−1^ OXC was prepared by dissolving 200 mg of pure OXC (Jubilant Organosys Ltd, Nanjangud, Mysore, India, purity 99.5%) in 40 mL of glacial acetic acid; the volume was brought to 100 mL with water in a volumetric flask and used for assay in method A. 

A 100 *μ*g mL^−1^ OXC standard solution was also prepared by dissolving an accurately weighed 10 mg of pure OXC in 10 mL of glacial acetic acid and diluting to the mark with water in a 100 mL calibrated flask, and this was diluted with 1 : 9 acetic acid to get a working concentration of 20 *μ*g mL^−1^ OXC and used in method B.

### 2.3. General Analytical Procedure

#### 2.3.1. Titrimetry (Method A)

A 3–9 mL aliquot of pure drug solution containing 6–18 mg of OXC was accurately measured and transferred into a 100 mL titration flask. The total volume was brought to 10 mL by adding 2 : 3 acetic acid. Ten mL of 10 M H_2_SO_4_ was added, and the solution titrated with 0.01 M NBS using a drop of methyl orange as indicator until the red colour disappeared. A blank titration was run and the necessary volume corrections were made. The amount of OXC in the measured aliquot was calculated from:

(1) $$\text{Amount}\;\left(\text{mg}\right)=\;VM_{w}\frac{R}{n},$$

where *V* = volume of NBS consumed, mL, *M*_*w*_ = relative molecular mass of drug (252.268), *R* = molar concentration of NBS, and *n* is the reaction stoichiometry (number of moles NBS reacting with each mole of OXC).

#### 2.3.2. Spectrophotometry (Method B)

Different aliquots (0.4–4.0 mL) of standard 20 *μ*g mL^−1^ OXC solution were transferred into a series of 10 mL volumetric flasks using a microburette, and the total volume in all the flasks was adjusted to 4 mL by adding 1 : 9 acetic acid. To each flask, 1 mL each of 10 M H_2_SO_4 _ and 120 *μ*g mL^−1^ NBS solutions were added, the content was mixed well and kept aside for 30 min at room temperature with occasional shaking. Finally, 1 mL of 0.03% BPR was added to each flask and the volume was made up to mark with water. Then, the absorbance of the unreacted dye was measured at 460 nm against reagent blank prepared similarly without OXC.

A standard graph was prepared by plotting absorbance against concentration, and the unknown concentration was read from the standard graph or computed from the regression equation derived using Beer's law data.

### 2.4. Procedure for the Analysis of Tablets

#### 2.4.1. Method A

Twenty tablets were weighed and finely powdered. An accurately weighed quantity of the tablet powder equivalent to 200 mg OXC was transferred into a 100 mL volumetric flask, and about 40 mL of glacial acetic acid was added. The content of the flask was shaken for 10 min, 30 mL of water was added, shaken for 10 more min, and finally the volume was completed to the mark with water. The content was mixed well and filtered through a Whatman no. 42 filter paper. First 10 mL portion of the filtrate was discarded and a suitable aliquot (say 5 mL) was then subjected to analysis by following the procedure described earlier.

#### 2.4.2. Method B

A quantity of tablet powder containing 10 mg of OXC was transferred into a 100 mL volumetric flask containing 10 mL of glacial acetic acid. The content was shaken for 5 min, 50 mL of water was added, and content was mixed well and shaken again for 15 more min. The mixture was diluted to the mark with water and filtered using Whatman no. 42 filter paper. First 10 mL portion of the filtrate was discarded and the resulting tablet extract (100 *μ*g mL^−1^ in OXC) was diluted to 20 *μ*g mL^−1^ with 1 : 9 acetic acid. Suitable aliquot was then subjected to analysis by following the general procedure.

### 2.5. Procedures for the Analysis of Placebo Blank and Synthetic Mixture

A placebo blank containing starch (10 mg), acacia (15 mg), hydroxyl cellulose (10 mg), sodium citrate (10 mg), talc (20 mg), magnesium stearate (15 mg) and sodium alginate (10 mg) was prepared by combining all components to form a homogeneous mixture. A 5 mg of the placebo blank was accurately weighed and its solution was prepared as described under “tablets”, and then subjected to analysis by following the general procedure.

A synthetic mixture was prepared by adding an accurately weighed 200 mg of OXC to the placebo mentioned above. The extraction procedure for tablets as described for method A and method B were applied separately by taking required quantity of synthetic mixture to prepare 2 mg mL^−1^ and 20 *μ*g mL^−1^ OXC solutions, respectively. Three different volumes of the resulting synthetic mixture solution (Equivalent to 6, 12 and 18 mg OXC in method A; 2, 4 and 6 *μ*g mL^−1^ in method B) were subjected to analysis by following the respective general procedure.

## 3. Chemistry

**Scheme 1:**
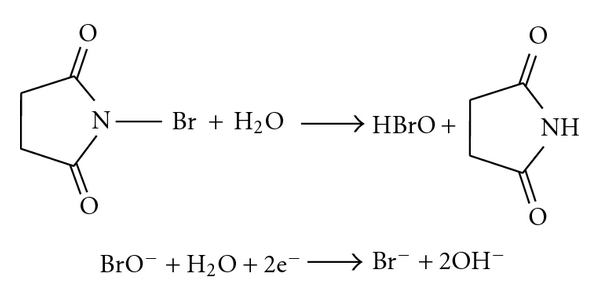
Hydrolysis of NBS to produce hypobromous acid/monovalent bromine.

N-Bromosuccinimide contains unstably bound bromine and is used for bromination in organic chemistry [22]. In the reactions where NBS used as brominating agent, the monovalent positive bromine of the NBS (the bond between bromine and nitrogen is polarized by the two neighbouring carbonyl groups) is responsible for bromination. The formation of the monovalent bromine due to hydrolysis of NBS is as shown in Scheme 1.

**Scheme 2:**
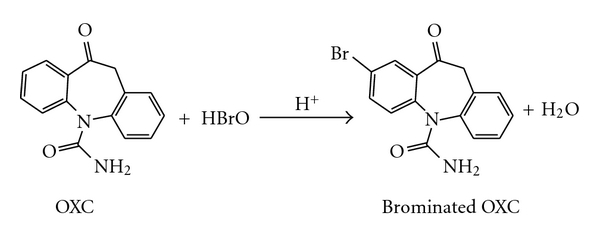
Suggested reaction pathway for the bromination of OXC.

The proposed methods are based on the bromination of the drug by NBS in sulphuric acid medium. The suggested reaction pathway for the bromination of OXC is given in Scheme 2.

**Figure 1:**
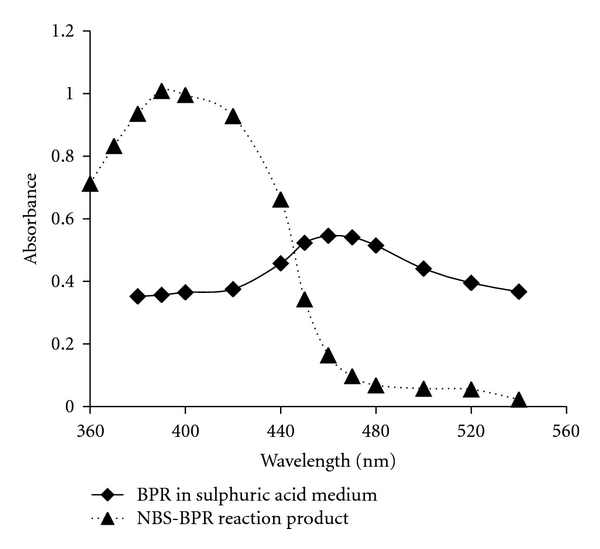
Absorption spectra of BPR in sulphuric acid medium and the reagent blank (bromoderivative of the dye, BPR).

**Scheme 3:**
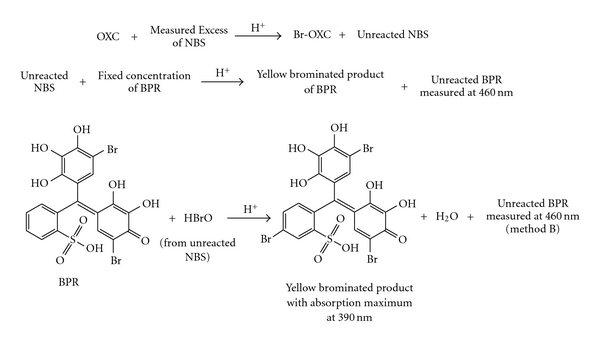
Tentative reaction pathway for the formation of yellow coloured bromo-derivative of BPR (method B).

In the proposed titrimetric method, OXC is directly titrated with NBS in sulphuric acid medium. The reaction between OXC and NBS was found to occur in 1 : 1 (drug : NBS) stoichiometric ratio, and all the calculations are based on this fact. Using 0.01 M NBS, 6–18 mg of OXC was conveniently determined. In spectrophotometry, a known excess of NBS was treated with OXC and after the reaction between OXC and NBS ensured to be complete, the unreacted NBS was reacted with a fixed concentration of BPR followed by the measurement of residual dye at 460 nm (Figure 1 and Scheme 3).

## 4. Results and Discussions

### 4.1. Method Development: Optimization of Experimental Variables

#### 4.1.1. Method A

A series of experiments was performed to select the solvent system to dissolve OXC. The drug is not soluble in any of the solvents like water, HCl, H_2_SO_4_, HNO_3_, and H_3_PO_4_ except acetic and perchloric acids. The titration was not feasible in perchloric acid medium. The titration was performed in the presence of different volumes and different concentrations of perchloric acid. No consistent stoichiometry was obtained at any volume/concentration of perchloric acid. At very high concentrations of perchloric acid, blank consumption was more, and at lower concentrations no reaction of OXC with NBS took place. The drug solution was found to be unstable when stored for longer time, and colloidal particles were observed when OXC was dissolved in acetic acid of concentration lower than 2 : 3 (acetic acid : water). Therefore, 2 : 3 acetic acid was used as solvent system.

Preliminary experiments were carried out to choose the proper medium for the quantitative and stoichiometric reaction between OXC and NBS. Sulphuric acid medium yielded a consistent stoichiometry with sharp end point. The reaction was rapid and quantitative when 8–12 mL of 10 M H_2_SO_4_ in a total volume of 20 mL was maintained. When the volume was less than 8 mL and greater than 12 mL, slightly lower and higher stoichiometric ratios, respectively, were observed. Therefore, 10 mL of 10 M H_2_SO_4_ was used throughout the investigation. Under the optimized reaction conditions, a definite reaction stoichiometry of 1 : 1 (OXC : NBS) was found for the range of 6–18 mg of OXC.

#### 4.1.2. Method B

**(1) Absorption Spectra**

BPR in H_2_SO_4_ medium is red in colour and exhibited an absorption maximum at 460 nm. Addition of BPR to NBS resulted in the formation of yellow coloured product (bromo derivative of BPR) with an absorption maximum at 390 nm. OXC and NBS had no absorption at either 460 or 390 nm. When increasing concentrations of OXC were reacted with a fixed concentration of NBS in acid medium, there occurred a concomitant decrease in the concentration of NBS. Addition of a fixed concentration of BPR to decreasing concentrations of NBS resulted in a proportional increase in the concentration of residual BPR leading to a linear increase in absorbance at 460 nm with the drug concentration, which formed the basis for the assay. Figure 1 illustrates the absorption spectra of BPR, and the reaction product formed between NBS and BPR in the absence of OXC (reagent blank).

**(2) Selection of Reaction Medium**

OXC solution in dilute acetic acid (1 : 9) is stable for longer time (more than two days) at 4°C. The reaction between OXC and NBS was performed in different acid media. Better results were obtained in sulphuric acid medium. The effect of acid concentration on the reaction between OXC and NBS was studied by varying the concentration of H_2_SO_4_ keeping the concentrations of NBS and drug fixed. The reaction was found to be rapid yielding a constant absorbance with maximum sensitivity and stability when the H_2_SO_4_ concentration was maintained in the range of 0.7–1.3 M (0.7 to 1.3 mL of 10 M in a total volume of 10 mL). The same acid concentration was found sufficient for the instantaneous reaction between unreacted NBS and BPR. At acid concentrations higher than 1.5 M, the solution becomes cloudy. Therefore, 1 mL of 10 M H_2_SO_4_ in a total volume of 10 mL was used throughout the work.

**(3) Optimization of NBS**

To fix the optimum concentration of NBS, different concentrations of NBS were reacted with a fixed concentration of BPR in H_2_SO_4_ medium and the absorbance was measured at 460 nm. A constant and minimum absorbance resulted with 12 *μ*g mL^−1^ NBS and, hence, different concentrations of OXC were reacted with 1 mL of 120 *μ*g mL^−1^ NBS in H_2_SO_4_ medium before determining the residual NBS *via* the reaction Scheme illustrated earlier. This facilitated the optimization of the linear dynamic range over which procedure could be applied for the assay of OXC.

**(4) Optimization of BPR**

To fix the upper Beer's law limit with respect to BPR, different concentrations of the dye were reacted with a fixed 1 mL of 120 *μ*g mL^−1^ NBS in sulphuric acid medium. After the reaction between NBS and BPR was ensured to be complete, the absorbance of the unreacted BPR was measured at 460 nm. It was found that a reproducible and minimum absorbance value was obtained when 1 mL of 0.03% BPR used. Hence, 1 mL of 0.03% of BPR was used throughout the investigation.

**(5) Study of Reaction Time and Stability of the Coloured Species**

**Figure 2:**
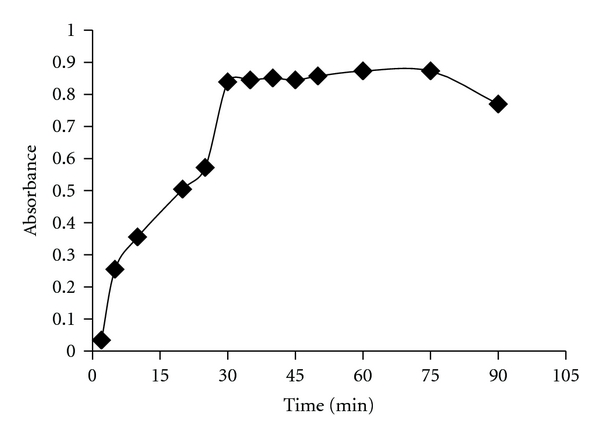
Study of reaction time between NBS and OXC in sulphuric acid medium and stability of the coloured species by measuring the absorbance of the unreacted BPR after reacting with NBS.

Under the described experimental conditions, the reaction between OXC and NBS was complete within 30 min (Figure 2) at room temperature (28 ± 2°C). After the addition of BPR, the reaction between NBS and dye was instantaneous and the absorbance of the unreacted dye was stable for at least 45 min, thereafter.

**(6) Effect of Diluent**

In order to select proper solvent for dilution, solvents like 1 : 9 acetic acid, water, methanol and 10 M H_2_SO_4_ were tried. Satisfactory results were obtained when water was used as the diluent.

### 4.2. Method Validation

#### 4.2.1. Linearity and Sensitivity

Over the range investigated (6–18 mg), a fixed reaction stoichiometry of 1 : 1 [OXC : NBS] was obtained in titrimetry which served as the basis for calculations. The relationship between the drug amount and the volume of titrant consumed was examined. The linearity between two parameters is apparent from the correlation coefficient of 0.9955 obtained by the method of least squares. From this, it is implied that the reaction between OXC and NBS proceeds stoichiometrically in the ratio 1 : 1 in the range studied. In spectrophotometry, the calibration graph was found to be linear from 0.8 to 8.0 *μ*g mL^−1^ OXC. The measured absorbance values were plotted *versus *concentration. The least square calibration equation was *A* = 0.1044C − 0.0113 [where the concentration (C) is measured in *μ*g mL^−1^] with a regression coefficient of 0.9993 (*n* = 5). The calculated molar absorptivity and Sandell sensitivity values are 2.52 × 10^4^ L mol^−1^ cm^−1^ and 0.010 *μ*g cm^−2^, respectively. The limits of detection (LOD) and quantification (LOQ) were calculated according to the ICH guidelines [23] using the formulae: 

LOD = 3.3 *S*/slope and LOQ = 10 *S*/slope, (where *S* is the standard deviation of the absorbance of six blank readings). The calculated LOD and LOQ are 0.28 and 0.86 *μ*g mL^−1^, respectively.

#### 4.2.2. Accuracy and Precision

The repeatability of the proposed methods was determined by performing replicate determinations (*n* = 7). The intraday and interday variation in the analysis of OXC was measured at three different levels. The accuracy of an analytical method expresses the closeness between the reference value and the found value. Accuracy was evaluated as percentage relative error between the measured and taken amounts/concentrations. The results of this study are compiled in Table 1 and speak of good intermediate precision (%RSD ≤ 3.06) and accuracy (%RE ≤ 3.75) of the results.

#### 4.2.3. Selectivity

In the analysis of placebo blank, the volume of NBS consumed was the same as that of the indicator blank (method A) and in spectrophotometry, the absorbance of the placebo blank was not different from that of the reagent blank suggesting the noninterference by the inactive ingredients added to prepare the placebo.

In method A, three different aliquots of the synthetic mixture extract were analyzed titrimetrically (*n* = 3 in each case), which yielded a % recovery values in the range from 98.66 to 103.1 of OXC with standard deviation values in the range of 0.62–1.74. In spectrophotometry, three different aliquots of 20 *μ*g mL^−1^ OXC were subjected to analysis (*n* = 3). The recovery values of 99.11–104.9% OXC with standard deviation 0.85−1.56 were obtained. These results which are close to 100% recovery complement the findings of the placebo blank analysis with respect to selectivity. The detailed results are presented in Table 2.

#### 4.2.4. Robustness and Ruggedness

To evaluate the robustness of the methods, volume of H_2_SO_4_ was slightly altered (10 ± 1 mL) with reference to optimum values in titrimetry. However, in spectrophotometry, the reaction time (after adding NBS, time varied was 30 ± 2 min) and volume of BPR (1 ± 0.2 mL of 0.03%) were slightly altered. To check the ruggedness, analysis was performed by four different analysts, and on three different burettes (method A) or spectrophotometers (method B) by the same analyst. The robustness and the ruggedness were checked at three different drug levels (6, 12, and 18 mg in method A; 2, 4, and 6 *μ*g mL^−1^ in method B). The intermediate precision, expressed as percent RSD, which is a measure of robustness and ruggedness, was within the acceptable limits (1.26–3.15%) as shown in Table 3.

#### 4.2.5. Application to Tablet Analysis

Commercial OXC tablets were analyzed using the developed methods and also by a reference published method [19]. The method is based on spectrophotometric determination of OXC using F-C reagent in alkaline medium. The results obtained were compared statistically by the Student's *t*-test and the variance-ratio *F*-test [24]. The calculated *t*- and *F*-values did not exceed the tabulated values of 2.77 (*t*) and 6.39 (*F*) at the 95 % confidence level and for four degrees of freedom, indicating close similarity between the proposed methods and the reference method with respect to accuracy and precision. These results are summarized in Table 4.

#### 4.2.6. Recovery Study

To further ascertain the accuracy and reliability of the methods, recovery experiments were performed *via* standard-addition procedure. Preanalyzed tablet powder was spiked (6 mg in method A; 2 *μ*g mL^−1^ in method B) with pure OXC at three different levels (3, 6, and 9 mg in method A; 1, 2, and 3 *μ*g mL^−1^ in method B), and the total was found by the proposed methods. Each determination was repeated three times. The percent recovery of pure OXC added (Table 5) was within the permissible limits indicating the absence of interference by the inactive ingredients in the assay.

## 5. Conclusions

One titrimetric and one new spectrophotometric method were developed and validated for the determination of oxcarbazepine using NBS as brominating agent. The titrimetric method, first to be reported for oxcarbazepine, is applicable over a semi-micro-scale (6–18 mg), and using spectrophotometry at a small concentration as 0.86 *μ*g mL^−1^ drug can be determined with confidence and with a fair degree of accuracy and precision. The new approach of utilizing NBS and bromopyrogallol red as reagents in spectrophotometry is the first of such reports. The method is the most sensitive ever developed for oxcarbazepine, though somewhat less rapid requiring a standing time of 30 min. Both methods are simple, accurate, and precise and are free from extreme experimental conditions such as heating at high temperature and use of organic solvent unlike in some reported methods. Both the methods were applied successfully to the determination of OXC in tablets. Compared to many existing instrumental methods for oxcarbazepine, the proposed spectrophotometric method has two additional advantages of simplicity of operations and low-cost instrument. Both the methods make use of very easily available and cheaper reagents which demonstrates their cost-effectiveness. These advantageous features advocate their use in quality control laboratories for routine use.

## Figures and Tables

**Table 1:** Intraday and interday accuracy and precision.

Method*	OXC taken	Intra-day accuracy and precision			Inter-day accuracy and precision		
		OXC found	RE%	RSD%	OXC found	RE%	RSD%
A	6.0	6.17	2.83	2.22	6.18	3.12	2.98
	12.0	12.28	2.33	1.98	11.73	2.22	3.06
	18.0	17.73	1.50	1.53	18.34	1.89	2.23

B	2.0	1.95	2.50	2.30	2.04	2.12	1.99
	4.0	4.07	1.75	1.20	3.94	1.52	1.58
	6.0	5.92	1.33	2.70	6.13	2.20	1.60

*In method A, OXC taken/found are in mg and they are *μ*g mL^−1^ in method B.

**Table 2:** Results obtained for the study of effect of matrix by the analysis of synthetic mixture.

Method*	OXC in synthetic mixture	OXC found^#^	%Recovery ± SD
A	6.0	5.92	98.66 ± 0.62
	12.0	12.07	100.6 ± 1.22
	18.0	18.56	103.1 ± 1.74

B	2.0	2.03	101.4 ± 1.56
	4.0	3.96	99.11 ± 0.85
	6.0	6.29	104.9 ± 1.14

*In method A, OXC taken/found are in mg and they are *μ*g mL^−1^ in method B.

^#^Mean value of three determinations.

**Table 3:** Robustness and ruggedness.

	Method A				Method B			
OXC studied mg	Robustness (RSD, %)	Ruggedness (RSD, %)		OXC studied *μ*g mL^−1^	Robustness (RSD, %)		Ruggedness (RSD, %)	
					Conditions altered*		Inter-analysts (*n* = 4)	Inter-instruments (*n* = 3)
	Volume of H_2_SO_4_ (*n* = 3)	Inter-analysis (*n* = 4)	Inter-burettes (*n* = 4)		Volume of BPR (*n* = 3)	Reaction time (*n* = 3)		

6.0	3.10	2.22	2.89	2.0	3.15	1.52	2.21	1.89
12.0	2.69	1.89	2.65	4.0	3.02	2.56	1.89	1.56
18.0	2.50	1.56	2.56	6.0	2.10	1.26	2.00	2.13

*In method A, volumes of 10 M H_2_SO_4_ varied were 10 ± 1 mL, In method B, volumes of BPR varied were 1 ± 0.1 mL, and the reaction time employed was 30 ± 2 min.

**Table 4:** Results of analysis of tablets by the proposed methods.

Tablets analysed	Label claim, mg/tablet	Found* (Percent of label claim ± SD)		
		Reference method	Method A	Method B
Trioptal 300	300	98.14 ± 1.56	97.33 ± 0.89	98.66 ± 1.12
			*t* = **1.04**	*t* = **0.61**
			*F* = **3.07**	*F* = **1.94**

Oxetol 600	600	102.1 ± 1.26	101.3 ± 1.56	100.2 ± 0.93
			*t* = **0.89**	*t* = **2.74**
			*F* = **1.53**	*F* = **1.84**

*Mean value of five determinations.

**Table 5:** Accuracy assessment by recovery experiments.

Tablets studied	Method A				Method B			
	OXC in tablet, mg	Pure OXC added,mg	Total found, mg	Pure OXC recovered*, Percent ± SD	OXC in tablet, *μ*g mL^−1^	Pure OXC added, *μ*g mL^−1^	Total found, *μ*g mL^−1^	Pure OXC recovered*, Percent ± SD
Oxetol 600	6.08	3.0	9.01	97.67 ± 0.56	2.00	1.00	3.02	101.6 ± 1.12
	6.08	6.0	12.43	105.83 ± 1.78	2.00	2.00	4.01	100.6 ± 0.88
	6.08	9.0	14.78	96.67 ± 0.58	2.00	3.00	5.14	104.5 ± 1.45

*Mean value of three determinations.
